# Body Weight Variability and Risk of Suicide Mortality: A Nationwide Population-Based Study

**DOI:** 10.1155/2024/7670729

**Published:** 2024-04-30

**Authors:** Jeongmin Lee, Jin-Hyung Jung, Dong Woo Kang, Min-Hee Kim, Dong-Jun Lim, Hyuk-Sang Kwon, Jung Min Lee, Sang-Ah Chang, Kyungdo Han, Seung-Hwan Lee

**Affiliations:** ^1^Division of Endocrinology and Metabolism, Department of Internal Medicine, Eunpyeong St. Mary's Hospital, College of Medicine, The Catholic University of Korea, Seoul, Republic of Korea; ^2^Samsung Biomedical Research Institute, Sungkyunkwan University School of Medicine, Suwon, Republic of Korea; ^3^Department of Psychiatry, Seoul St. Mary's Hospital, College of Medicine, The Catholic University of Korea, Seoul, Republic of Korea; ^4^Division of Endocrinology and Metabolism, Department of Internal Medicine, Seoul St. Mary's Hospital, College of Medicine, The Catholic University of Korea, Seoul, Republic of Korea; ^5^Division of Endocrinology and Metabolism, Department of Internal Medicine, Yeouido Mary's Hospital, College of Medicine, The Catholic University of Korea, Seoul, Republic of Korea; ^6^Department of Statistics and Actuarial Science, Soongsil University, Seoul, Republic of Korea; ^7^Department of Medical Informatics, College of Medicine, The Catholic University of Korea, Seoul, Republic of Korea

## Abstract

**Background:**

Suicide is a pressing global health concern, and identifying its risk factors is crucial for prevention. Body weight variability (BWV) has been increasingly recognized as a potential factor impacting physical and mental health outcomes. We aimed to explore the relationship between BWV and the risk of suicide mortality using a nationally representative database.

**Methods:**

This population-based cohort study used data from the Korean National Health Insurance Database and included a total of 1,983,701 subjects. BWV was assessed using at least three health examination datasets and validated variability indices (variability independent of the mean (VIM), average successive variability, and coefficient of variation), and patients were divided into BWV quartiles (Q1–Q4). The primary endpoint was suicide-related death.

**Results:**

During a median of 11.3 years of follow-up, 5,883 suicide deaths occurred. A higher baseline body weight was associated with a lower risk of suicide. However, greater BWV (VIM) was associated with a significantly greater risk of suicide (adjusted hazard ratio [95% confidence interval], 1.35 [1.26–1.45] in the Q4 group), even after adjusting for baseline body mass index (BMI). Similar results were observed regardless of obesity or BMI category. Consistent findings were observed when using different variability indices. Subgroup analyses according to sex, age, diabetes, and depression also supported these findings.

**Conclusion:**

Our study highlights the importance of considering BWV as a potential risk factor for suicide.

## 1. Introduction

Death from self-harm, especially suicide, is a significant global health issue, with nearly one million suicide-related deaths occurring worldwide each year [1]. Korea's suicide rate ranks as the highest among the Organization for Economic Cooperation and Development countries at 25.7 per 100,000 persons [2]. Intentional self-harm is the leading cause of death among individuals aged 10–19, 20–29, or 30–39 years and the second cause among those 40–49 or 50–59 years [1]. Suicide is widely recognized as a phenomenon that does not occur in isolation but rather because of a multifaceted process. Therefore, to prevent suicide, it is essential to identify the risk factors associated with suicidal death. Individuals who have made suicide attempts have a greater probability of suffering from psychiatric diseases like major depressive disorder, bipolar disorder, anxiety disorder, schizophrenia, and sleep disorders such as insomnia [3, 4]. In addition to psychiatric disorders, chronic diseases significantly increase the risk of suicide in both older and younger patients with enduring health conditions [5, 6]. Obesity is currently one of the most widespread chronic diseases, yet the independent connection between an increase in body weight (BW) and suicide rates remains uncertain. Previous population-based studies have confirmed that obesity independently increases the risk of suicide behaviors such as suicidal ideation, attempts, and death [7, 8]. In terms of body mass index (BMI), there seems to be a paradoxical inverse association with the risk of suicide [9, 10]. Recent research has also shown that patients who undergo bariatric surgery face an increased risk of death from suicide in the period following the procedure [11]. It was suggested that degrees from changes in BW, including inadequate weight loss and/or weight regain, could be associated with psychological function [12]. In fact, high BW variability (BWV) was associated with an increased risk of depression in both the general population and patients with type 2 diabetes mellitus (DM) [13, 14]. Nevertheless, there have been no prior investigations into the potential connection between BWV and suicide.

Our hypothesis was that greater BWV would be associated with an increased risk of suicide death in the general population, including individuals with psychiatric disorders. Consequently, our aim was to explore the relationship between BWV, assessed using validated variability indices and obesity status, and the risk of suicide mortality using repetitive health examinations and a claims database.

## 2. Materials and Methods

### 2.1. Data Collection and Participants

This was a retrospective cohort study performed using the database of the Korean National Health Insurance Service (NHIS). South Korea has a single and universal insurance system that provides healthcare coverage for nearly the entire population. All enrollees in the NHIS are advised to receive medical checkups at least every two years. The number of eligible individuals and actual examinees has increased gradually, and approximately 15 million people participate in health examinations every year. The rate of general health screening was approximately 75% in the last ten years [15]. The NHIS database is composed of a qualification database (including sex, age, income, area of residence, and types of qualification), a claim database (containing information such as specifications, consultation statements, diagnosis statements defined by the International Classification of Diseases 10th revision (ICD-10), and prescription statements), a health checkup database (including general health examination results and questionnaire answers on lifestyle and behavior), and death information [15].

Among 4,234,415 participants aged ≥ 20 years who underwent health examinations in 2009 (baseline year), a total of 2,250,714 subjects were excluded, including those with fewer than three health examinations in the previous five years (2005–2009) (*n* = 1,974,030), those with missing data (*n* = 272,763), and those who died within one year after the health screening (*n* = 9,921), to reduce the effect of reverse causality. Ultimately, a total of 1,983,701 subjects were analyzed and followed from baseline to December 2021. This study was approved by the Institutional Review Board of The Catholic University of Korea, Eunpyeong St. Mary's Hospital (no. PC23ZISI0150). Researchers can use the NHIS database after approval of the study protocol by the official review committee. The need for written informed consent was waived due to the use of previously collected data with unidentifiable information.

### 2.2. Measurements and Definitions of Covariates

Demographic and lifestyle data were obtained using a standardized self-reported questionnaire for the following variables: smoking status, alcohol consumption, and regular exercise (>30 min of moderate physical activity ≥ 5 times per week or >20 min of strenuous physical activity ≥ 3 times per week). Household income was divided into two categories based on the lower 25%. BW, height, and waist circumference (WC) were measured at each visit. BMI was calculated as weight in kilograms divided by the square of height in meters (kg/m^2^). Obesity was defined as BMI ≥ 25 kg/m^2^ based on the World Health Organization's recommendation for Asian populations (“Appropriate body-mass index for Asian populations and its implications for policy and intervention strategies,” 2004) [16]. Systolic blood pressure (SBP) and diastolic blood pressure (DBP) were measured in a seated position after ≥ 5 min of rest. Blood chemistry tests for glucose and lipid parameters were measured after overnight fasting. The criteria for DM were either a fasting glucose level ≥ 126 mg/dL or at least one annual medical claim under the ICD-10 codes E11–14 and the prescription of antidiabetic medication. Hypertension was determined as the SBP/DBP ≥ 140/90 mmHg or at least one claim per year under ICD-10 codes I10–13 or I15 and the prescription of antihypertensive agents. Dyslipidemia was defined as a serum total cholesterol (TC) level ≥ 240 mg/dL or at least one claim per year for prescription of lipid-lowering agents under ICD-10 code E78. Chronic kidney disease (CKD) was defined as an estimated glomerular filtration rate (eGFR) < 60 mL/min/1.73 m^2^, which was calculated using the modification of diet in renal disease formula, as follows: 186 × (serum creatinine) − 1.154 × age − 0.203 × 0.742 (if female). Psychiatric disorders were defined based on the ICD-10 code as follows: schizophrenia (F20), bipolar disorders (F30 or F31), depressive disorder (F32 or F33), anxiety (F40 or F41), and sleep disorder (F510).

#### 2.2.1. Definition of BWV

We calculated the within-individual visit-to-visit variability in BW using data collected from each health examination, requiring a minimum of three BW measurements performed over a five-year period, including the index date. Three measures of BWV were employed as follows: variability independent of the mean (VIM), coefficient of variation (CV), and average successive variability (ASV). VIM is calculated as 100 times the ratio of the standard deviation (SD) to the mean, with beta representing the regression coefficient derived from the natural logarithm of SD divided by the natural logarithm of the mean, providing a measure of variability independent of the mean BW. The CV is defined as the ratio of SD to the mean and provides a better reflection of variability since it is less influenced by the mean. Unlike VIM, CV directly compares variability relative to the mean BW [17]. The ASV is determined by calculating the average of the absolute differences between consecutive BW measurements. It provides a direct measure of variability between successive measurements, reflecting short-term fluctuations in BW [18].

#### 2.2.2. Definition of Study Outcomes

The primary study endpoint was the occurrence of suicide-related deaths during the follow-up period. The classification of death was based on verification of the date of death, and deaths by suicide were identified by confirming the presence of ICD-10 code X60–X84 based on the Korean Standard Classification of Disease, using the National Statistics Organization database [19].

### 2.3. Statistical Analysis

Continuous variables are presented as mean ± SD, and categorical variables are presented as number and percentage. For comparisons among groups of VIM, CV, or ASV quartiles of BW, the chi-square test was used for categorical variables, and analysis of variance was used for continuous variables. The incidence rate (IR) of death from suicide was calculated as the number of events divided by the total follow-up duration (1,000 person-years). The cumulative incidence of suicide according to the BWV quartiles was calculated using Kaplan–Meier curves, and the log-rank test was performed to analyze differences among the groups. Multivariable Cox proportional hazard models were used to estimate hazard ratios (HRs) and their corresponding 95% confidence intervals (CIs) to assess the risk of death from suicide considering BWV. We adjusted for age, sex, income, lifestyle factors, comorbidities, psychiatric diseases, and BMI or WC as appropriate. We also examined potential effect modification by obesity or BMI category. A sensitivity analysis was performed after excluding subjects with known psychiatric disorders. For the subgroup analysis, participants were categorized according to sex, age, DM, and depression, and interaction testing was performed using a likelihood ratio test. Statistical analyses were performed using SAS version 9.4 (SAS Institute Inc., Cary, NC, USA), and *P* < 0.05 was considered statistically significant.

## 3. Results

### 3.1. Baseline Characteristics of Participants according to BWV

The mean age and BMI of participants were 47.3 ± 13.3 years and 23.8 ± 3.1 kg/m^2^, respectively. Comparisons of baseline characteristics among BWV groups based on VIM are demonstrated in Table 1. Males were more common, and subjects were older in low-variability groups. Although the *P* values for trend were <0.001 for all variables due to the large size of the study population, the BMI, WC, SBP, DBP, glucose, and TC were similar across groups. Obesity was more frequent in Q1 and Q4 than in Q2 and Q3 groups. The presence of depression, bipolar disease, schizophrenia, anxiety, and insomnia, respectively, was greatest in the Q4 BWV group. Q1 BWV participants were more likely to engage in regular exercise compared to those with greater BWV, and a current smoking habit was the least common in the Q1 BWV group.

### 3.2. Risk of Suicide Mortality according to BW

In the entire cohort, 110,158 (5.6%) events of death and 5,883 (0.3%) events of death from suicide occurred during a median of 11.3 years of follow-up. Both all-cause death and death from suicide were most frequent in the Q4 BWV group (Table 1). An analysis was performed to evaluate the association between BW quartiles at index date and suicidal risk, as presented (Supporting Information: Table S1). As BW increased from Q1 to Q4, there was a significant decrease in suicide death risk (adjusted hazard ratio (aHR) [95% CI], 0.77 [0.71–0.83] in Q2 vs. 0.63 [0.58–0.68] in Q3 vs. 0.53 [0.49–0.58] in Q4).

### 3.3. Risk of Suicide Mortality according to BWV

**Figure 1:**
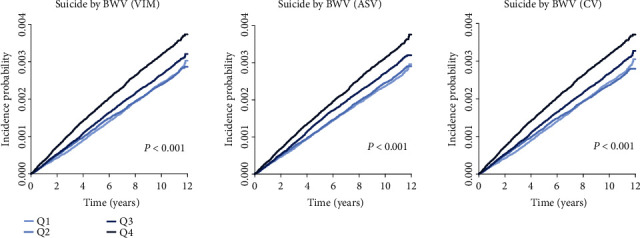
Kaplan–Meier estimates of cumulative incidence of suicide death according to the variability of body weight. ASV: average successive variability; BWV: body weight variability; CV: coefficient of variation; VIM: variability independent of the mean.

Table 2 presents the results of suicide death risk based on BWV during follow-up. Compared to the reference group with the lowest BWV (Q1), individuals with greater BWV demonstrated significantly high suicidal risks. A progressive increase in both the IR and HR for suicide death across the quartiles of BWV was observed. In the Q3 and Q4 BWV groups, the risk of suicide death increased by 14% and 39%, respectively, following adjustment for age, sex, income, alcohol drinking, smoking, regular exercise, comorbidities, and psychiatric disorders (aHR [95% CI], 1.14 [1.06–1.23] in Q3 BWV and 1.39 [1.29–1.49] in Q4 BWV). Similar results were maintained after further adjustment for BMI among Q3 BWV participants (aHR [95% CI], 1.12 [1.04–1.20]), with a further increase for Q4 BWV participants (aHR [95% CI] 1.35 [1.26–1.45]). When adjusting for WC instead of BMI, the results remained similar (data not shown). When adjusted using different criteria for dyslipidemia, defined as a serum triglyceride (TG) level ≥ 200 mg/dL, low-density lipoprotein cholesterol level (LDL‐C) ≥ 160 mg/dL, high-density lipoprotein cholesterol level (HDL‐C) < 40 mg/dL, or at least one annual prescription claims for lipid-lowering agents under the ICD-10 code E78 [20], the results remained consistent (data not shown). Similar results were observed when using other BWV indices, such as ASV (aHR [95% CI], 1.11 [1.03–1.19] in the Q3 group and 1.29 [1.20–1.38] in the Q4 group) and CV (1.13 [1.05–1.21] in the Q3 group and 1.34 [1.25–1.44] in the Q4 group). Kaplan–Meier estimates of cumulative incidence showed similar patterns (Figure 1).

### 3.4. The Association between Risk of Suicide Mortality and BWV according to Obesity and BMI Category

**Figure 2:**
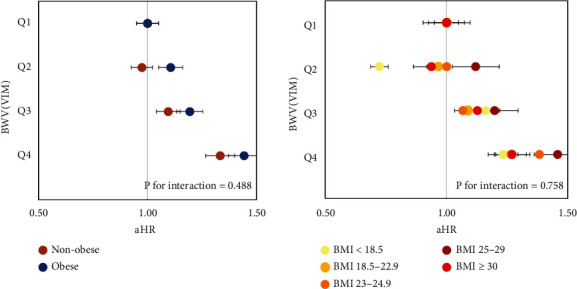
The association between BWV (VIM) and risk of suicide death in different obesity or BMI categories. Obesity was defined as BMI ≥ 25 kg/m^2^. aHR: adjusted hazard ratio; BMI: body mass index; BWV: body weight variability; VIM: variability independent of the mean. Age, sex, income, lifestyle factors, comorbidities, psychiatric diseases, and waist circumference were adjusted.

Further analyses were performed to evaluate the relationship between BWV and the risk of suicide death in different obesity or BMI categories (Figure 2). Regardless of obesity, greater BWV was associated with a significantly high aHR (aHR [95% CI] in the Q4 BWV group, 1.33, [1.22–1.45] in nonobese vs. 1.44 [1.27–1.65] in obese participants; *P* for interaction = 0.488). Similarly, the risk of suicide death was significantly increased in the Q3 and Q4 BWV groups regardless of BMI category. The BWV Q4 group showed the highest aHRs, which ranged from 1.27 to 1.46 among normal-weight, overweight, and obese participants. The risk of suicide death was highest in the group of patients with BMI 25–30 kg/m^2^ (aHR [95% CI], 1.46 [1.27–1.67]). These results were consistent when applying the different BWV indices with ASV and CV (Supporting Information: Figure S1).

### 3.5. Sensitivity Analysis

Because the presence of psychiatric diseases is a significant risk factor for suicide, we conducted a sensitivity analysis after excluding participants with depression, bipolar disease, schizophrenia, anxiety, and insomnia (Supporting Information: Table S2). The trends in IR, HR, and aHR for suicide death aligned with the results from the original analysis. Notably, the risk of suicide was 9%–11% higher in the Q3 BWV group (aHR [95% CI], 1.09 [1.01–1.18] by VIM vs. 1.11 [1.02–1.20] by ASV vs. 1.10 [1.02–1.20] by CV) and 28%–32% higher in the Q4 BWV group (aHR [95% CI], 1.32 [1.21–1.42] by VIM vs. 1.28 [1.18–1.39] by ASV vs. 1.31 [1.21–1.42] by CV) compared to rates in the Q1 BWV group.

### 3.6. Subgroup Analyses

We performed subgroup analyses according to sex, age, DM, and depression (Table 3, Supporting Information: Figure S2). The IR of suicide was approximately three times greater in men than in women (IR, 0.43 vs. 0.16 per 1,000 person-years in the Q4 group). A similar pattern of an increase in HR according to higher variability was present for both sexes (aHR of the male Q4 BWV group, 1.35 [1.24–1.46] vs. that of the female group, 1.37 [1.16–1.61]; *P* for interaction = 0.842). The IR of death by suicide was highest among individuals aged > 65 years. The pattern of increase in aHR with greater BWV was consistent across age groups (*P* for interaction = 0.657). DM was identified as a risk factor for suicide mortality compared to non-DM (IR, 0.30/1,000 person-years in the non-DM Q4 BWV group vs 0.49/1,000 person-years in the DM Q4 BWV group). However, the increase in aHR with high BWV was not significant in the DM group, while the aHR was significantly increased with high BWV in the non-DM group (*P* for interaction = 0.027). People with depression had much higher IRs compared to people without depression. The increase in aHR according to BWV was steeper in people with depression, although a significant difference was not noted (*P* for interaction = 0.321). The above data, which were analyzed using VIM as the variability index, were replicated when using ASV or CV (Supporting Information: Figures S3 and S4).

We also performed subgroup analyses according to the fasting blood glucose levels and lipid (LDL-C, HDL-C, and TG) levels. Higher quartiles of BWV (Q3 and Q4) were significantly associated with an elevated risk of suicide compared to the reference group, particularly among individuals with normal fasting glucose and impaired fasting glucose levels. However, lipid levels did not modify the association between body weight variability and suicide (Supporting Information: Table S3).

Last, we conducted an analysis to examine whether the relationship between BWV and risk of suicide death differs according to baseline BW (Supporting Information: Table S4). In this analysis, individuals with high BW (Q4) and high BWV (Q4) exhibited the highest aHR at 1.47 (95% CI, 1.27–1.72). The BWV in the Q4 category was identified as a risk factor for suicide, irrespective of the baseline BW (*P* for interaction = 0.938).

## 4. Discussion

In this large nationwide population-based study with nearly two million participants, we showed a linear relationship between BWV, assessed by three distinct validated variability indices (VIM, ASV, and CV), and the risk of suicide mortality. Importantly, this relationship persisted even after adjusting for baseline BMI and remained regardless of obesity or BMI subgrouping. Our study reveals significant findings regarding the association between BWV and the risk of death from suicide, providing novel insight into the risk and prevention of suicide.

According to our findings, baseline BW was inversely correlated with the risk of suicide. Individuals in the highest quartile (Q4) showed a 47% reduced risk of suicide (aHR [95% CI], 0.53 [0.49–0.58]) compared to those in the lowest quartile. This result aligns with the results of previous studies using several cohorts. A study conducted in Sweden revealed that, for each 5 kg/m^2^ increase in BMI, the risk of suicide decreased by 15% [9]. Similarly, a study using data from the U.S. National Health Interview Surveys demonstrated that, with each 5 kg/m^2^ increase in BMI, the risk of suicide decreased by 18% for men and 24% for women [21]. Previous retrospective cohort studies and analyses have reported an inverse relationship between obesity and suicide mortality (completed suicide) [22, 23]. Several possible mechanisms for the association between BW and suicide have been proposed. A high BMI is suggested to be associated with reduced impulsive suicide attempts by virtue of its correlation with various hormones and neurotransmitters. Reduced level of central serotonin is consistently linked to heightened impulsivity [24]. In obese individuals, insulin resistance is associated with elevated postprandial free fatty acid (FFA) level [25]. These FFAs compete with tryptophan, the precursor of serotonin, for binding to serum albumin. Consequently, the increase in FFAs leads to elevated levels of both free tryptophan and serotonin. Hence, the elevation in serotonin in obese individuals may be linked to a lower risk of suicide [26]. Serum leptin demonstrates a positive correlation with BMI, suggesting a compensatory mechanism for severe leptin resistance [27]. Leptin also has immunomodulatory properties. Chronic inflammation has been linked to mood disorders, including depression, and is a risk factor for suicide. Leptin's potential to reduce inflammation might indirectly lower the risk of suicidal ideation and behavior. Leptin has been implicated in neuroprotection and neuroplasticity and promotes the survival and growth of neurons in the brain, which can impact mood and cognitive function [28]. However, the potential links between obesity or BMI and established risk factors for completed suicide have not been thoroughly examined.

In our study, the highest BWV (Q4) was associated with a 35% increased risk of death by suicide compared to that in the lowest quartile (Q1), irrespective of obesity. This pattern was similar in both men and women. The IR of completed suicide was approximately three times higher in men than in women. In the general population, there are approximately 1.5 times the number of suicide attempts by men, and approximately 3.5 times more males die by suicide [29], which is similar to our data. In a subgroup analysis categorized by age, the greatest BWV led to the highest aHR irrespective of age group. However, the IR of completed suicide was highest among individuals with the greatest BWV and those aged > 65 years. Elderly adults were consistently found to be at a higher risk for suicide compared to other age groups in nearly all countries, according to the World Health Organization (“Appropriate body-mass index for Asian populations and its implications for policy and intervention strategies,” 2004). Several factors are associated with an elevated risk of suicide in older adults, including psychiatric illnesses (especially depression), cooccurring physical illnesses, a history of prior suicide attempts, limited social support, and difficulty coping with stressors [30]. Beyond the direct causal effect between BWV and metabolic disturbances, BWV might indicate difficulty in maintaining homeostasis and potentially contribute to a decline in mental well-being. There have been a few studies that have reported on the association between BW and mental health conditions, like dementia [31] and depression [14]. However, until recently, there has been no research investigating the relationship between BWV and suicidal behavior, including suicide death. One study investigated an association between BW change and suicide [32]. Unexplained weight loss was associated with a high risk of suicide (HR [95% CI], 2.48 [1.04–5.92]) in men. While the underlying mechanism remains unclear, there are several potential pathways. Prolonged and greater BWV could potentially trigger a chronic neuroinflammatory response [33]. Increased levels of inflammatory cytokines such as interleukin- (IL-) 4 and IL-10 could lead to changes in motivation and emotion [34]. Several cross-sectional studies demonstrated that high levels of IL-6 were correlated with a high risk of suicide [35, 36]. Long-term BWV interferes with glucose homeostasis, resulting in increased triglyceride and glucose levels [37]. The relationship observed between insulin resistance and suicidal behavior was explained by cytokine-mediated inflammation, which triggers tryptophan depletion, and the onset of suicidal behavior associated with serotonergic dysfunction, depression, and impulsivity [38]. Lipid variability in BWV is another plausible mechanism. A meta-analysis with 65 epidemiologic studies explored a potential connection between low serum cholesterol levels and a high risk of suicide [39]. Changes in cholesterol levels could impact lipid raft activity and alter synaptic transmission and neural plasticity, leading to the development of brain disorders [40].

Loss of energy, reduced appetite, and BW loss are prevalent symptoms included in the diagnostic criteria for depression. However, bidirectional influences exist between depression and appetite or weight dysregulation. Atypical depression, a subtype distinguished by hypersomnia, psychomotor slowing, increased mood reactivity, and interpersonal rejection sensitivity, is characterized by features such as increased appetite and weight gain [41]. The abovementioned immunometabolic abnormalities have been proposed as contributors to the change of appetite and BWV in depression. Patients with depression may relapse episodically without adequate treatment. BWV itself may involve repeated weight loss during depressive episodes followed by subsequent weight regain in the recovery phase, or vice versa. Hence, higher BWV may intensify suicide death, indicating a potential link to uncontrolled depression and these outcomes.

Numerous promising antiobesity medications, including semaglutide and tirzepatide, are currently available. Nevertheless, their low adoption, ineffectiveness, and discontinuation may be associated with insufficient weight loss or regain. These complexities within treatment dynamics may contribute to increase BWV. The lack of treatment success and subsequent weight regain could exacerbate stigmatization, and the psychological stress from the failure of antiobesity medications emphasizes the importance of carefully considering mental health aspects in obesity treatment.

To our knowledge, this is the first study to investigate the association between BWV and the risk of death from suicide among the general population. This study is based on a large sample size using nationwide data, enhancing the reliability of our results. To ensure the accuracy of our findings, we controlled potential confounding variables, including comorbidities, psychiatric disorders, and baseline BMI. However, several limitations should be acknowledged. Because the rate of general health screening was approximately 75%, the results cannot be equally applied to subjects who did not undergo health screenings. Due to the design of the study, a causal relationship or direction of effects between BWV and suicidal risk could not be confirmed. To reduce the possibility of reverse causation, we excluded people with outcomes within the first year of follow-up. We could not differentiate between intentional weight loss and unintentional weight fluctuations as our study primarily relied on a health examination database. As a result, we were unable to establish specific correlations between intentional or unintentional weight changes and the risk of suicide. Prospective and longitudinal research is needed to investigate this association and to reveal potential mechanisms. Additionally, since our study focused on the Korean population, our findings might not be generalizable to populations of diverse ethnic origins. The validity of lifestyle variables derived from self-reported questionnaires in the study may be influenced by several factors. Denial or underreporting of drinking or smoking could occur due to social desirability bias. However, despite these limitations, the utilization of such simple methods is deemed a reasonable alternative to more extensive questionnaires in large-scale surveys. We recognize that some unmeasured factors, such as the severity of psychopathology or treatment of psychiatric disorders, could have influenced the outcomes. However, we adjusted for common psychological conditions related to suicide based on diagnostic codes. Moreover, our results remained similar even after excluding psychiatric disorders, suggesting that the associations were not confounded by preexisting mental problems. Lastly, this study limited the classification of suicide mortality to ICD-10 codes X60–X84, excluding cases categorized as undetermined deaths. Consequently, there is a possibility of underestimating suicides.

## 5. Conclusion

Our study highlights a significant and previously underexplored relationship between higher BWV and increased risk of suicide mortality. This association persisted irrespective of obesity status or sex and was particularly pronounced in non-DM subjects. Overall, our study adds to the growing body of evidence supporting the negative effect of BWV on mental health, including completed suicide. Future studies are needed to confirm our findings and to explore the underlying mechanisms by which BWV may affect suicide.

## Figures and Tables

**Table 1:** Baseline characteristics of participants according to the body weight variability (VIM).

Parameters	Total (*n* = 1983701)	Body weight variability			
		Q1 (*n* = 495353)	Q2 (*n* = 496832)	Q3 (*n* = 495595)	Q4 (*n* = 495921)
Male	1241020 (62.6)	317761 (64.2)	319121 (64.2)	314771 (63.5)	289367 (58.4)
Age (years)	47.3 ± 13.3	48.6 ± 12.6	47.5 ± 12.6	46.9 ± 13.1	46.1 ± 14.7
Height (cm)	164.9 ± 9.1	165.0 ± 8.9	165.1 ± 9.0	165.1 ± 9.1	164.5 ± 9.5
Weight (kg)	64.9 ± 11.5	65.1 ± 10.9	64.7 ± 11.2	64.9 ± 11.4	64.8 ± 12.5
WC (cm)	80.7 ± 8.8	81.1 ± 8.7	80.6 ± 8.7	80.6 ± 8.7	80.7 ± 9.2
BMI (kg/m^2^)	23.8 ± 3.1	23.8 ± 3.0	23.7 ± 3.0	23.7 ± 3.1	23.8 ± 3.4
Systolic BP (mmHg)	122.6 ± 14.5	123.0 ± 14.4	122.6 ± 14.4	122.6 ± 14.4	122.4 ± 14.7
Diastolic BP (mmHg)	76.7 ± 9.8	76.8 ± 9.9	76.7 ± 9.8	76.7 ± 9.8	76.4 ± 9.8
Glucose (mg/dL)	97.0 ± 22.7	97.2 ± 21.0	96.8 ± 21.5	96.8 ± 22.5	97.1 ± 25.4
TC (mg/dL)	195.7 ± 36.2	196.3 ± 35.9	195.8 ± 35.8	195.7 ± 36.1	194.9 ± 37.0
Obesity	653706 (33.0)	169986 (34.3)	157806 (31.8)	159286 (32.1)	166628 (33.6)
Diabetes	163479 (8.2)	40710 (8.2)	38904 (7.8)	39830 (8.0)	44035 (8.9)
Hypertension	496898 (25.1)	130998 (26.5)	122664 (24.7)	121243 (24.5)	121993 (24.6)
Dyslipidemia	343453 (17.3)	88361 (17.8)	84937 (17.1)	84502 (17.1)	85653 (17.3)
CKD	144518 (7.3)	37287 (7.5)	36070 (7.3)	34894 (7.0)	36267 (7.3)
Low income	322203 (16.2)	79251 (16.0)	79605 (16.0)	80115 (16.2)	83232 (16.8)
Depression	54817 (2.8)	12017 (2.4)	12035 (2.4)	13434 (2.7)	17331 (3.5)
Bipolar disease	1493 (0.08)	215 (0.04)	251 (0.05)	378 (0.08)	649 (0.13)
Schizophrenia	1783 (0.09)	281 (0.06)	306 (0.06)	421 (0.08)	775 (0.16)
Anxiety	116568 (5.9)	27612 (5.6)	27330 (5.5)	28747 (5.8)	32879 (6.6)
Insomnia	55537 (2.8)	12979 (2.6)	12500 (2.5)	13487 (2.7)	1657 (3.3)
Current smoking	550233 (27.7)	131963 (26.6)	139310 (28.0)	142887 (28.8)	136073 (27.4)
Alcohol drinking	1029575 (51.9)	259989 (52.5)	263717 (53.1)	260864 (52.6)	245005 (49.4)
Regular exercise	380953 (19.2)	99848 (20.2)	97467 (19.6)	94698 (19.1)	88940 (17.9)
All-cause death	110158 (5.6)	24198 (4.9)	23502 (4.7)	25976 (5.2)	36482 (7.4)
Suicide	5883 (0.30)	1347 (0.27)	1340 (0.27)	1473 (0.30)	1723 (0.35)

Data are expressed as mean ± SD, or *n* (%). *P* values for the trend were <0.001 for all variables due to the large size of the study population. BMI: body mass index; BP: blood pressure; CKD: chronic kidney disease; TC: total cholesterol; VIM: variability independent of the mean; WC: waist circumference.

**Table 2:** The risk of suicide according to the quartiles of body weight variability.

Variability	*N*	Events	Incidence rate⁣^∗^	Model 1	Model 2	Model 3	Model 4
VIM							
Q1	495353	1347	0.24	1 (reference)	1 (reference)	1 (reference)	1 (reference)
Q2	496832	1340	0.24	0.99 (0.92-1.07)	1.03 (0.95-1.11)	1.02 (0.94-1.10)	1.01 (0.94-1.09)
Q3	495595	1473	0.27	1.10 (1.02-1.18)	1.16 (1.08-1.25)	1.14 (1.06-1.23)	1.12 (1.04-1.20)
Q4	495921	1723	0.32	1.30 (1.21-1.40)	1.46 (1.36-1.56)	1.39 (1.29-1.49)	1.35 (1.26-1.45)
*P* for trend				<0.001	<0.001	<0.001	<0.001
ASV							
Q1	540752	1440	0.24	1 (reference)	1 (reference)	1 (reference)	1 (reference)
Q2	477320	1315	0.25	1.03 (0.56-1.11)	1.01 (0.94-1.09)	1.01 (0.93-1.09)	1.02 (0.95-1.10)
Q3	468980	1424	0.27	1.15 (1.07-1.23)	1.11 (1.03-1.19)	1.08 (1.00-1.16)	1.11 (1.03-1.19)
Q4	496649	1704	0.31	1.30 (1.22-1.40)	1.29 (1.20-1.38)	1.22 (1.14-1.31)	1.29 (1.20-1.38)
*P* for trend				<0.001	<0.001	<0.001	<0.001
CV							
Q1	495895	1358	0.25	1 (reference)	1 (reference)	1 (reference)	1 (reference)
Q2	495524	1319	0.24	0.97 (0.90-1.10)	1.01 (0.94-1.09)	1.01 (0.93-1.08)	0.99 (0.92-1.07)
Q3	496139	1489	0.27	1.10 (1.02-1.18)	1.17 (1.10-1.26)	1.15 (1.07-1.24)	1.13 (1.05-1.21)
Q4	496143	1717	0.32	1.28 (1.19-1.38)	1.46 (1.36-1.57)	1.39 (1.29-1.49)	1.34 (1.25-1.44)
*P* for trend				<0.001	<0.001	<0.001	<0.001

⁣^∗^Per 1000 person-years. Model 1: unadjusted. Model 2: adjusted for age, sex, income, alcohol drinking, smoking, and regular exercise. Model 3: adjusted for model 2 plus diabetes, hypertension, dyslipidemia, chronic kidney disease, depression, bipolar disease, schizophrenia, anxiety, and insomnia. Model 4: adjusted for model 3 plus body mass index. ASV: average successive variability; CV: coefficient of variation; VIM: variability independent of the mean.

**Table 3:** The risk of suicide according to the quartiles of body weight variability (VIM) in subgroups by sex, age, diabetes, and depression.

	*N*	Events	Incidence rate⁣^∗^	HR (95% CI)	Adjusted HR (95% CI)^#^
*Sex*					
Male					
Q1	317761	1121	0.32	1 (reference)	1 (reference)
Q2	319121	1111	0.31	0.99 (0.91-1.07)	1.01 (0.93-1.10)
Q3	314771	1228	0.35	1.11 (1.02-1.20)	1.13 (1.04-1.23)
Q4	289367	1354	0.43	1.35 (1.25-1.46)	1.35 (1.24-1.46)
Female					
Q1	177592	226	0.11	1 (reference)	1 (reference)
Q2	177711	229	0.12	1.01 (0.84-1.22)	1.02 (0.85-1.23)
Q3	180824	245	0.12	1.07 (0.89-1.28)	1.06 (0.89-1.28)
Q4	206554	369	0.16	1.42 (1.20-1.68)	1.37 (1.16-1.62)
*P* for interaction				0.790	0.842
*Age*					
20-39 years					
Q1	129241	247	0.17	1 (reference)	1 (reference)
Q2	146774	299	0.18	1.07 (0.90-1.26)	1.07 (0.90-1.26)
Q3	164735	340	0.18	1.08 (0.92-1.27)	1.10 (0.93-1.29)
Q4	200734	434	0.19	1.13 (0.97-1.33)	1.30 (1.11-1.52)
40-64 years					
Q1	305882	753	0.22	1 (reference)	1 (reference)
Q2	296387	750	0.23	1.03 (0.93-1.14)	1.02 (0.92-1.13)
Q3	274631	789	0.26	1.17 (1.06-1.29)	1.16 (1.05-1.28)
Q4	225361	784	0.31	1.43 (1.29-1.58)	1.40 (1.26-1.54)
≥65 years					
Q1	60230	347	0.55	1 (reference	1 (reference)
Q2	53671	291	0.52	0.95 (0.81-1.11)	0.94 (0.80-1.09)
Q3	56229	344	0.60	1.08 (0.93-1.26)	1.03 (0.89-1.20)
Q4	69826	505	0.74	1.35 (1.17-1.54)	1.22 (1.06-1.40)
*P* for interaction				0.053	0.657
*Diabetes*					
No					
Q1	454643	1156	0.23	1 (reference)	1 (reference)
Q2	457928	1158	0.23	1.00 (0.92-1.08)	1.01 (0.93-1.10)
Q3	455765	1303	0.26	1.13 (1.04-1.22)	1.15 (1.07-1.25)
Q4	451886	1498	0.30	1.32 (1.22-1.42)	1.40 (1.29-1.51)
Yes					
Q1	40710	191	0.43	1 (reference)	1 (reference)
Q2	38904	182	0.43	1.00 (0.81-1.22)	1.01 (0.82-1.24)
Q3	39830	170	0.40	0.92 (0.75-1.13)	0.92 (0.75-1.13)
Q4	44035	225	0.49	1.13 (0.93-1.37)	1.10 (0.90-1.33)
*P* for interaction				0.143	0.027
*Depression*					
No					
Q1	483336	1272	0.24	1 (reference)	1 (reference)
Q2	484797	1247	0.23	0.98 (0.90-1.06)	1.00 (0.92-1.08)
Q3	482161	1349	0.25	1.07 (0.99-1.15)	1.10 (1.02-1.19)
Q4	478590	1528	0.29	1.23 (1.14-1.32)	1.33 (1.24-1.43)
Yes					
Q1	12017	75	0.57	1 (reference)	1 (reference)
Q2	12035	93	0.71	1.24 (0.91-1.68)	1.23 (0.91-1.67)
Q3	13434	124	0.85	1.49 (1.12-1.98)	1.44 (1.08-1.91)
Q4	17331	195	1.07	1.87 (1.44-2.44)	1.68 (1.28-2.19)
*P* for interaction				0.024	0.321

⁣^∗^Per 1000 person-years. ^#^Adjusted for age, sex, income, smoking, drinking, regular exercise, diabetes, hypertension, dyslipidemia, chronic kidney disease, depression, bipolar disease, schizophrenia, anxiety, insomnia, and body mass index. CI: confidence interval; VIM: variability independent of the mean.

## Data Availability

All data generated or analyzed during this study are included in this published article and its Supporting Information files. The data can be accessed on the National Health Insurance Data Sharing Service homepage of the NHIS (https://nhiss.nhis.or.kr).

## Supplementary Materials

Supplementary Materials Figure S1 (a): the association between body weight variability (ASV) and risk of suicide death in different obesity or BMI categories. We conducted an evaluation to assess the relationship between body weight variability based on average successive variability (ASV) and the risk of suicide death in various obesity or BMI categories. Regardless of obesity, greater BWV was associated with a significantly higher adjusted hazard ratio (aHR) ([95% CI] in the Q4 BWV group, 1.27 [1.72–1.39] in nonobese vs. 1.30 [1.14–1.49] in obese participants; *P* for interaction = 0.257). The risk of suicide death was highest in the group of patients with a BMI of 23–25 kg/m^2^ (aHR [95% CI], 1.35 [1.17–1.55]). Figure S1 (b): the association between body weight variability (CV) and risk of suicide death in different obesity or BMI categories. We evaluated the connection between body weight variability, specifically focusing on coefficient of variation (CV), and the risk of suicide death across various obesity or BMI categories. Irrespective of obesity status, a higher body weight variability (BWV) demonstrated a notably elevated adjusted hazard ratio (aHR) ([95% CI] in the Q4 BWV group, 1.22 [0.86–1.74] for nonobese individuals compared to 1.40 [1.23–1.60] for obese participants; *P* for interaction = 0.560). Notably, the group with a BMI of 25–30 kg/m^2^ exhibited the highest risk of suicide death (aHR [95% CI], 1.42 [1.24–1.63]). Figure S2: the relationship between BWV (VIM) and risk of suicide death according to sex, age, DM, and depression. Figure S3: the relationship between BWV (ASV) and risk of suicide death according to sex, age, DM, and depression. Figure S4: the relationship between BWV (CV) and risk of suicide death according to sex, age, DM, and depression. The incidence rate of suicide was approximately three times higher in men than in women (IR, 0.43 vs. 0.16 per 1,000 person-years in the Q4 group). Both sexes exhibited a similar pattern of increased hazard ratio (aHR) with higher variability (aHR of the male Q4 BWV group, 1.35 [1.24–1.46] vs. that of the female group, 1.37 [1.16–1.61]; *P* for interaction = 0.842). For individuals aged > 65 years, the IR of death by suicide was the highest, and the pattern of increased aHR with greater BWV was consistent across age groups (*P* for interaction = 0.657). DM was identified as a risk factor for suicide mortality (IR, 0.3/1,000 person-years in the non-DM Q4 BWV group vs. 0.49/1,000 person-years in the DM Q4 BWV group). However, the increase in aHR with high BWV was not significant in the DM group, while it was significantly increased in the non-DM group (*P* for interaction = 0.027). Individuals with depression had much higher IRs compared to those without depression. The increase in aHR according to BWV was steeper in people with depression, although a significant difference was not noted (*P* for interaction = 0.321). These findings, using VIM as the variability index, were consistent when using ASV or CV (Supporting Information: Figures S3 and S4). Table S1: the risk for suicide according to the quartile of baseline body weight. An analysis was conducted to assess the association between body weight (BW) quartiles at the index date and suicidal risk, revealing a significant decrease in suicide death risk with increasing BW from Q1 to Q4 (adjusted hazard ratio (aHR) [95% CI]: 0.77 [0.71–0.83] in Q2, 0.63 [0.58–0.68] in Q3, and 0.53 [0.49–0.58] in Q4). Table S2: the risk of suicide according to the quartiles of body weight variability (sensitivity analysis after excluding mental diseases). A sensitivity analysis, excluding participants with psychiatric diseases such as depression, bipolar disease, schizophrenia, anxiety, and insomnia reaffirmed the trends observed in the original analysis. Notably, the risk of suicide showed a 9%–11% increase in the Q3 BWV group (aHR [95% CI]: 1.09 [1.01–1.18] by VIM, 1.11 [1.02–1.20] by ASV, and 1.10 [1.02–1.20] by CV) and a 28%–32% increase in the Q4 BWV group (aHR [95% CI]: 1.32 [1.21–1.42] by VIM, 1.28 [1.18–1.39] by ASV, and 1.31 [1.21–1.42] by CV) compared to rates in the Q1 BWV group. Table S3: the risk for suicide according to the quartiles of baseline body weight and body weight variability. We investigated whether the association between body weight variability (BWV) and the risk of suicide death varied based on baseline body weight (Supporting Information: Table S3). Notably, individuals with both high baseline body weight (Q4) and high BWV (Q4) showed the highest adjusted hazard ratio (aHR) at 1.47 (95% CI, 1.27–1.72). Importantly, BWV in the Q4 category emerged as a consistent risk factor for suicide, independent of baseline body weight (*P* for interaction = 0.938). Table S4: the risk of suicide according to the quartiles of body weight variability (VIM) in subgroups by serum glucose level and lipid. Higher quartiles of BWV (Q3 and Q4) were significantly associated with an elevated risk of suicide compared to the reference group, particularly among individuals with normal fasting glucose and impaired fasting glucose levels. Regarding lipid levels, the analysis revealed a significant association between BWV (Q4); however, there was no significant difference based on lipid levels.
