# Supplementary material for: Body Weight Variability and Risk of Suicide Mortality: A Nationwide Population-Based Study
Source: Depress Anxiety. 2024 Apr 30;2024:7670729. doi: 10.1155/2024/7670729 (PMC11921691; doi:10.1155/2024/7670729)
Supplement: Supplementary Materials — Figure S1 (a): the association between body weight variability (ASV) and risk of suicide death in different obesity or BMI categories. We conducted an evaluation to assess the relationship between body weight variability based on average successive variability (ASV) and the risk of suicide death in various obesity or BMI categories. Regardless of obesity, greater BWV was associated with a significantly higher adjusted hazard ratio (aHR) ([95% CI] in the Q4 BWV group, 1.27 [1.72–1.39] in nonobese vs. 1.30 [1.14–1.49] in obese participants; P for interaction = 0.257). The risk of suicide death was highest in the group of patients with a BMI of 23–25 kg/m2 (aHR [95% CI], 1.35 [1.17–1.55]). Figure S1 (b): the association between body weight variability (CV) and risk of suicide death in different obesity or BMI categories. We evaluated the connection between body weight variability, specifically focusing on coefficient of variation (CV), and the risk of suicide death across various obesity or BMI categories. Irrespective of obesity status, a higher body weight variability (BWV) demonstrated a notably elevated adjusted hazard ratio (aHR) ([95% CI] in the Q4 BWV group, 1.22 [0.86–1.74] for nonobese individuals compared to 1.40 [1.23–1.60] for obese participants; P for interaction = 0.560). Notably, the group with a BMI of 25–30 kg/m2 exhibited the highest risk of suicide death (aHR [95% CI], 1.42 [1.24–1.63]). Figure S2: the relationship between BWV (VIM) and risk of suicide death according to sex, age, DM, and depression. Figure S3: the relationship between BWV (ASV) and risk of suicide death according to sex, age, DM, and depression. Figure S4: the relationship between BWV (CV) and risk of suicide death according to sex, age, DM, and depression. The incidence rate of suicide was approximately three times higher in men than in women (IR, 0.43 vs. 0.16 per 1,000 person-years in the Q4 group). Both sexes exhibited a similar pattern of increased hazard ratio (aHR) with highe [file 7670729.f1.zip › Supplementary figures and tables.docx]

**Supplementary Material**

**The association between risk of suicide mortality and body weight variability according to obesity and body mass index category (Figure S1 (a) and Figures S1 (b))**


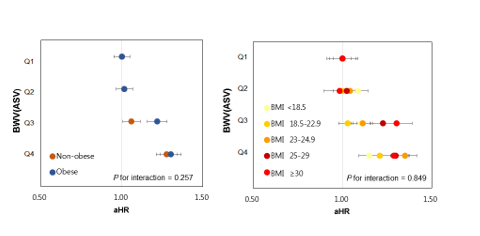
 **Figure S1 (a)** The association between body weight variability (ASV) and risk of suicide death in different obesity or BMI categories

aHR, adjusted hazard ratio; ASV, average successive variability; BMI, body mass index; BWV, body weight variability


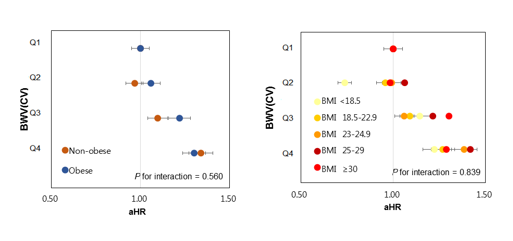


**Figure S1 (b)** The association between body weight variability (CV) and risk of suicide death in different obesity or BMI categories

aHR, adjusted hazard ratio; BMI, body mass index; BWV, body weight variability; CV, coefficient of variation

**Subgroup analyses according to sex, age, dabetes mellitus, and depression (Figure S2, Figure S3, Figure S4).**


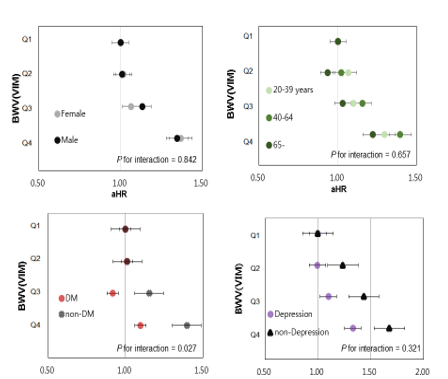


**Figure S2.** The relationship between BWV (VIM) and risk of suicide death according to sex, age, DM, and depression.

aHR, adjusted hazard ratio; BWV, body weight variability; DM, diabetes mellitus; VIM, variability independent of the mean


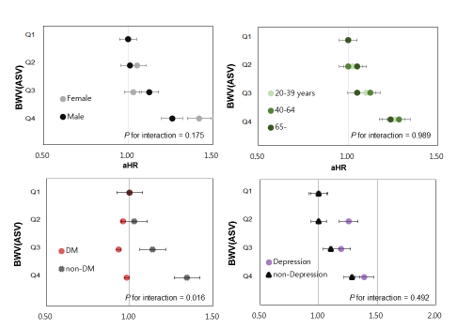


**Figure S3.** The relationship between BWV (ASV) and risk of suicide death according to sex, age, DM, and depression.

aHR, adjusted hazard ratio; ASV, average successive variability; BWV, body weight variability; DM, diabetes mellitus


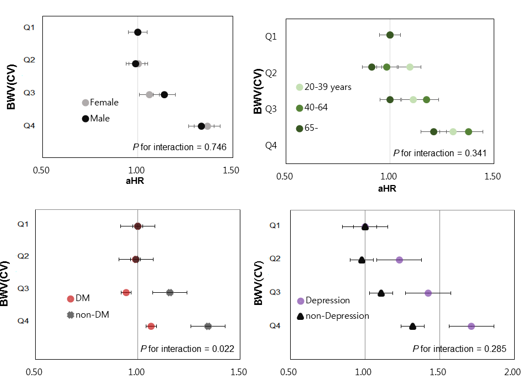


**Figure S4.** The relationship between BWV (CV) and risk of suicide death according to sex, age, DM, and depression.

aHR, adjusted hazard ratio; BWV, body weight variability; DM, diabetes mellitus; CV, coefficient of variation

**Table S1.** The risk for suicide according to the quartile of baseline body weight

| **Baseline body weight** | **N** | **Events** | **Incidence rate*** | **HR (95% CI)** | | |
| --- | --- | --- | --- | --- | --- | --- |
|  |  |  |  | **Model 1** | **Model 2** | **Model 3** |
| Q1 | 503787 | 1363 | 0.25 | 1 (Reference) | 1 (Reference) | 1 (Reference) |
| Q2 | 512153 | 1663 | 0.29 | 1.20 (1.11-1.28) | 0.78 (0.72-0.84) | 0.77 (0.71-0.83) |
| Q3 | 480438 | 1546 | 0.29 | 1.18 (1.10-1.27) | 0.64 (0.59-0.70) | 0.63 (0.58-0.68) |
| Q4 | 487323 | 1311 | 0.24 | 0.98 (0.91-1.06) | 0.56 (0.51-0.61) | 0.53 (0.49-0.58) |
| *P* for trend |  |  |  | 0.616 | <0.001 | <0.001 |

*per 1000 person-years

Model 1: Unadjusted

Model 2: Adjusted for age, sex, income, alcohol drinking, smoking, and regular exercise

Model 3: Adjusted for model 2 plus diabetes, hypertension, dyslipidemia, chronic kidney disease, depression, bipolar disease, schizophrenia, anxiety, and insomnia

| **BW Variability** | **N** | | **Events** | **Incidence rate*** | **HR (95% CI)** | | | |
| --- | --- | --- | --- | --- | --- | --- | --- | --- |
|  |  |  |  |  | **Model 1** | **Model 2** | **Model 3** | **Model 4** |
| **VIM** | |  |  |  |  |  |  |  |
| Q1 | | 451060 | 1143 | 0.23 | 1 (Reference) | 1 (Reference) | 1 (Reference) | 1 (Reference) |
| Q2 | | 453393 | 1092 | 0.21 | 0.95 (0.88-1.03) | 0.98 (0.90-1.06) | 0.98 (0.90-1.06) | 0.97 (0.89-1.05) |
| Q3 | | 449485 | 1195 | 0.24 | 1.05 (0.97-1.14) | 1.11 (1.02-1.20) | 1.11 (1.02-1.20) | 1.09 (1.01-1.18) |
| Q4 | | 442204 | 1316 | 0.27 | 1.19 (1.10-1.29) | 1.35 (1.25-1.46) | 1.34 (1.24-1.45) | 1.32 (1.21-1.42) |
| P for trend | |  |  |  | <0.001 | <0.001 | <0.001 | <0.001 |
| **ASV** | |  |  |  |  |  |  |  |
| Q1 | | 489553 | 1186 | 0.22 | 1 (Reference) | 1 (Reference) | 1 (Reference) | 1 (Reference) |
| Q2 | | 437936 | 1095 | 0.22 | 1.03 (0.95-1.12) | 1.00 (0.92-1.08) | 0.99 (0.92-1.08) | 1.01 (0.95-1.10) |
| Q3 | | 422708 | 1143 | 0.24 | 1.12(1.03-1.22) | 1.08 (0.99-1.17) | 1.07 (0.99-1.16) | 1.11 (1.02-1.20) |
| Q4 | | 445945 | 1322 | 0.27 | 1.24 (1.14-1.34) | 1.22 (1.12-1.31) | 1.21 (1.14-1.31) | 1.28 (1.18-1.39) |
| P for trend | |  |  |  | <0.001 | <0.001 | <0.001 | <0.001 |
| **CV** | |  |  |  |  |  |  |  |
| Q1 | | 451965 | 1153 | 0.23 | 1 (Reference) | 1 (Reference) | 1 (Reference) | 1 (Reference) |
| Q2 | | 452311 | 1075 | 0.21 | 0.93 (0.86-1.01) | 0.96 (0.89-1.05) | 0.96 (0.89-1.05) | 0.95 (0.86-1.03) |
| Q3 | | 450115 | 1212 | 0.24 | 1.06 (0.98-1.15) | 1.12 (1.04-1.22) | 1.12 (1.04-1.22) | 1.10 (1.02-1.20) |
| Q4 | | 441751 | 1306 | 0.27 | 1.17 (1.08-1.27) | 1.35 (1.25-1.57) | 1.35 (1.24-1.46) | 1.31 (1.21-1.42) |
| *P* for trend | |  |  |  | <0.001 | <0.001 | <0.001 | <0.001 |

**Table S2.** The risk of suicide according to the quartiles of body weight variability (Sensitivity analysis after excluding mental diseases)

*per 1000 person-years

Model 1: Unadjusted

Model 2: Adjusted for age, sex, income, alcohol drinking, smoking, and regular exercise

Model 3: Adjusted for model 2 plus diabetes, hypertension, dyslipidemia, and chronic kidney disease

Model 3: Adjusted for model 3 plus body mass index

ASV, average successive variability; BW, body weight; CV, coefficient of variation; VIM, variability independent of the mean

**Table S3**. The risk of suicide according to the quartiles of body weight variability (VIM) in subgroups by serum glucose level and lipid

|  | **N** | **Events** | **Incidence rate*** | **HR (95% CI)** | **Adjusted HR (95% CI)^#^** |
| --- | --- | --- | --- | --- | --- |
| **FG level (<100 mg/dL)** | | | | | |
| Q1 | 334118 | 796 | 0.21 | 1 (Reference) | 1 (Reference) |
| Q2 | 340991 | 846 | 0.22 | 1.04 (0.95-1.15) | 1.06 (0.96-1.17) |
| Q3 | 341819 | 917 | 0.24 | 1.13 (1.03-1.24) | 1.16 (1.05-1.27) |
| Q4 | 343401 | 1058 | 0.28 | 1.31 (1.19-1.43) | 1.39 (1.27-1.53) |
| **FG level ( 100-125 mg/dL)** | | | | | |
| Q1 | 120525 | 360 | 0.27 | 1 (Reference) | 1 (Reference) |
| Q2 | 116937 | 312 | 0.24 | 0.89 (0.77-1.04) | 0.91 (0.78-1.05) |
| Q3 | 113946 | 386 | 0.31 | 1.14 (0.99-1.31) | 1.15 (1.00-1.32) |
| Q4 | 108485 | 440 | 0.37 | 1.38 (1.20-1.59) | 1.42 (1.23-1.63) |
| **FG level (126 ≥mg/dL)** | | | | | |
| Q1 | 40710 | 191 | 0.434 | 1 (Reference) | 1 (Reference) |
| Q2 | 38904 | 182 | 0.433 | 1.00 (0.81-1.22) | 1.01 (0.82-1.24) |
| Q3 | 39830 | 170 | 0.398 | 0.92 (0.75-1.13) | 0.92 (0.75-1.13) |
| Q4 | 44035 | 225 | 0.492 | 1.13 (0.93-1.37) | 1.10 (0.90-1.33) |
| *P* for interaction |  |  |  | 0.070 | 0.031 |
| **LDL-C level (<160 mg/dL)** | | | | | |
| Q1 | 450594 | 1250 | 0.25 | 1 (Reference) | 1 (Reference) |
| Q2 | 453290 | 1231 | 0.24 | 0.98 (0.91-1.06) | 1.00 (0.93-1.09) |
| Q3 | 452234 | 1356 | 0.27 | 1.08 (1.00-1.17) | 1.12 (1.03-1.21) |
| Q4 | 453315 | 1573 | 0.32 | 1.27 (1.18-1.37) | 1.35 (1.25-1.45) |
| **LDL-C level (≥160 mg/dL)** | | | | | |
| Q1 | 44759 | 97 | 0.19 | 1 (Reference) | 1 (Reference) |
| Q2 | 43542 | 109 | 0.22 | 1.16 (0.88-1.512) | 1.18 (0.90-1.55) |
| Q3 | 43361 | 117 | 0.24 | 1.25 (0.95-1.63) | 1.28(0.98-1.67) |
| Q4 | 42606 | 150 | 0.32 | 1.65 (1.28-2.13) | 1.70 (1.32-2.20) |
| *P* for interaction |  |  |  | 0.291 | 0.383 |
| **TG (<200 mg/dL)** | | | | | |
| Q1 | 408066 | 1046 | 0.23 | 1 (Reference) | 1 (Reference) |
| Q2 | 410857 | 1067 | 0.23 | 1.01 (0.93-1.10) | 1.03 (0.95-1.12) |
| Q3 | 411284 | 1169 | 0.26 | 1.11 (1.02-1.21) | 1.13 (1.04-1.23) |
| Q4 | 414918 | 1357 | 0.30 | 1.29 (1.19-1.40) | 1.34 (1.23-1.45) |
| **TG (≥200 mg/dL)** | | | | | |
| Q1 | 87287 | 301 | 0.31 | 1 (Reference) | 1 (Reference) |
| Q2 | 85975 | 273 | 0.29 | 0.92 (0.78-1.09) | 0.94 (0.80-1.11) |
| Q3 | 84311 | 304 | 0.32 | 1.05 (0.89-1.23) | 1.08 (0.92-1.27) |
| Q4 | 81003 | 366 | 0.41 | 1.33 (1.14-1.55) | 1.43 (1.23-1.66) |
| *P* for interaction |  |  |  | 0.527 | 0.345 |
| **HDL-C (≥40 mg/dL)** | | | | | |
| Q1 | 436020 | 1167 | 0.24 | 1 (Reference) | 1 (Reference) |
| Q2 | 438743 | 1168 | 0.24 | 1.00 (0.92-1.08) | 1.01 (0.93-1.10) |
| Q3 | 438456 | 1292 | 0.27 | 1.10 (1.021.20) | 1.13 (1.04-1.22) |
| Q4 | 440948 | 1501 | 0.31 | 1.29 (1.19-1.39) | 1.35 (1.25-1.46) |
| **HDL-C (<40 mg/dL)** | | | | | |
| Q1 | 59333 | 180 | 0.27 | 1 (Reference) | 1 (Reference) |
| Q2 | 58089 | 172 | 0.27 | 0.97 (0.79-1.20) | 1.01 (0.82-1.24) |
| Q3 | 57139 | 181 | 0.29 | 1.05 (0.85-1.29) | 1.09 (0.88-1.33) |
| Q4 | 54973 | 222 | 0.37 | 1.36 (1.12-1.66) | 1.38 (1.13-1.68) |
| *P* for interaction |  |  |  | 0.780 | 0.960 |

*per 1000 person-years

#Adjusted for age, sex, income, smoking, drinking, regular exercise, diabetes, hypertension, dyslipidemia, chronic kidney disease, depression, bipolar disease, schizophrenia, anxiety, insomnia, and body mass index

CI, confidence interval; FG, fasting glucose; HDL-C, high-density lipoprotein cholesterol; LDL-C, low-density lipoprotein cholesterol; TG, triglyceride; VIM, variability independent of the mean

|  | **N** | **Events** | **Incidence rate*** | **HR (95% CI)** | | | |
| --- | --- | --- | --- | --- | --- | --- | --- |
|  |  |  |  | **Model 1** | **Model 2** | **Model 3** | **Model 4** |
| **Baseline BW Q1** |  |  |  |  |  |  |  |
| VIM Q1 | 113304 | 255 | 0.20 | 1 (Reference) | 1 (Reference) | 1 (Reference) | 1 (Reference) |
| VIM Q2 | 128613 | 293 | 0.20 | 1.01 (0.86-1.20) | 0.98 (0.83-1.16) | 0.98 (0.83-1.15) | 0.98 (0.83-1.16) |
| VIM Q3 | 124497 | 336 | 0.24 | 1.21 (1.03-1.42) | 1.11 (0.94-1.30) | 1.08 (0.92-1.27) | 1.09 (0.92-1.28) |
| VIM Q4 | 137373 | 479 | 0.32 | 1.60 (1.37-1.86) | 1.38 (1.18-1.60) | 1.29 (1.11-1.50) | 1.27 (1.09-1.48) |
| **Baseline BW Q2** |  |  |  |  |  |  |  |
| VIM Q1 | 132699 | 387 | 0.26 | 1 (Reference) | 1 (Reference) | 1 (Reference) | 1 (Reference) |
| VIM Q2 | 125692 | 386 | 0.28 | 1.05 (0.92-1.21) | 1.04 (0.91-1.21) | 1.04 (0.90-1.20) | 1.04 (0.90-1.20) |
| VIM Q3 | 131850 | 426 | 0.29 | 1.11 (0.97-1.28) | 1.14 (0.99-1.31) | 1.12 (0.97-1.28) | 1.11 (0.97-1.28) |
| VIM Q4 | 121912 | 464 | 0.35 | 1.32 (1.16-1.52) | 1.42 (1.24-1.63) | 1.35 (1.18-1.55) | 1.34 (1.17-1.53) |
| **Baseline BW Q3** |  |  |  |  |  |  |  |
| VIM Q1 | 127737 | 404 | 0.28 | 1 (Reference) | 1 (Reference) | 1 (Reference) | 1 (Reference) |
| VIM Q2 | 124842 | 365 | 0.26 | 0.92 (0.80-1.06) | 0.95 (0.83-1.10) | 0.95 (0.82-1.10) | 0.94 (0.82-1.10) |
| VIM Q3 | 115979 | 370 | 0.29 | 1.01 (0.88-1.16) | 1.08 (0.94-1.24) | 1.06 (0.92-1.22) | 1.05 (0.91-1.21) |
| VIM Q4 | 111880 | 407 | 0.33 | 1.16 (1.10-1.33) | 1.37 (1.20-1.58) | 1.30 (1.13-1.50) | 1.30 (1.13-1.49) |
| **Baseline BW Q4** |  |  |  |  |  |  |  |
| VIM Q1 | 121613 | 301 | 0.22 | 1 (Reference) | 1 (Reference) | 1 (Reference) | 1 (Reference) |
| VIM Q2 | 117685 | 296 | 0.23 | 1.01 (0.95-1.19) | 1.07 (0.91-1.26) | 1.07 (0.91-1.25) | 1.07 (0.91-1.26) |
| VIM Q3 | 123269 | 341 | 0.25 | 1.12 (0.96-1.30) | 1.23 (1.06-1.44) | 1.21 (1.04-1.41) | 1.22 (1.05-1.43) |
| VIM Q4 | 124756 | 373 | 0.27 | 1.21 (1.04-1.41) | 1.50 (1.29-1.75) | 1.44 (1.24-1.68) | 1.47 (1.27-1.72) |
| *P* for interaction |  |  |  | 0.080 | 0.984 | 0.974 | 0.938 |

**Table S4**. The risk for suicide according to the quartiles of baseline body weight and body weight variability

*per 1000 person-years

Model 1: Unadjusted

Model 2: Adjusted for age, sex, income, alcohol drinking, smoking, and regular exercise

Model 3: Adjusted for model 2 plus diabetes, hypertension, dyslipidemia, chronic kidney disease, depression, bipolar disease, schizophrenia, anxiety, and insomnia

Model 4: Adjusted for model 3 plus body mass index

BW, body weight; VIM, variability independent of the mean
