# Supplementary figures and images for: Body Weight Variability and Risk of Suicide Mortality: A Nationwide Population-Based Study
Source: Depress Anxiety. 2024 Apr 30;2024:7670729. doi: 10.1155/2024/7670729 (PMC11921691; doi:10.1155/2024/7670729)

Figure S1.(A)

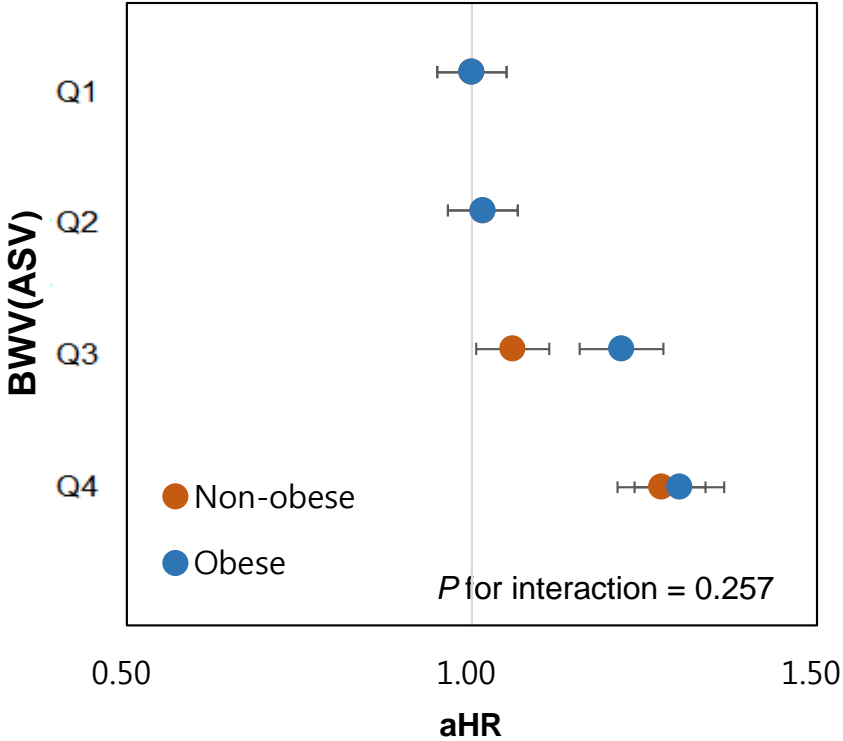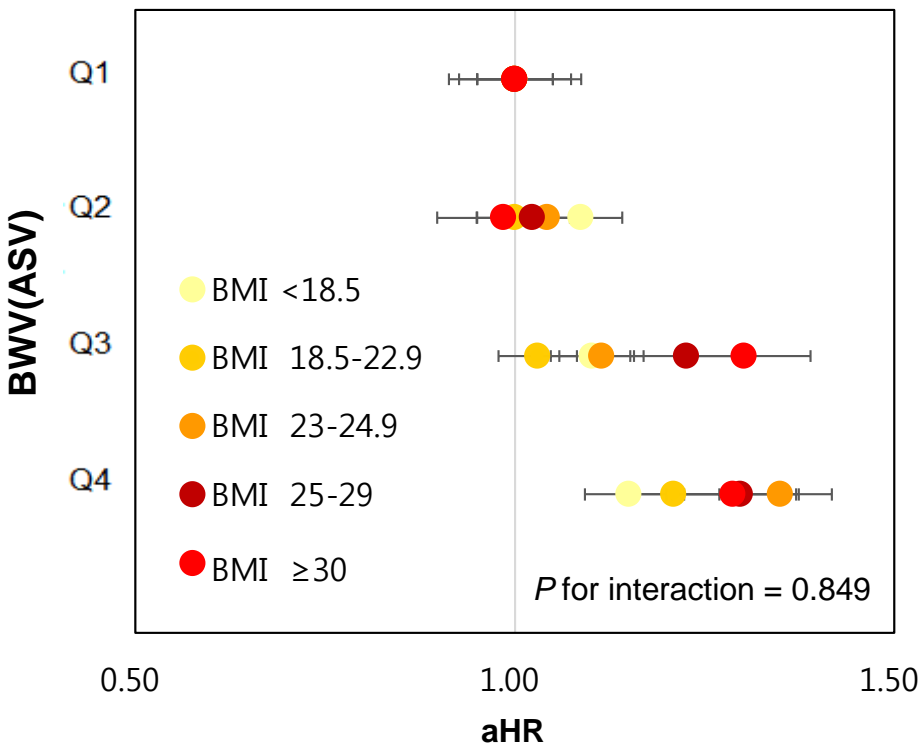

Figure S1.(B)

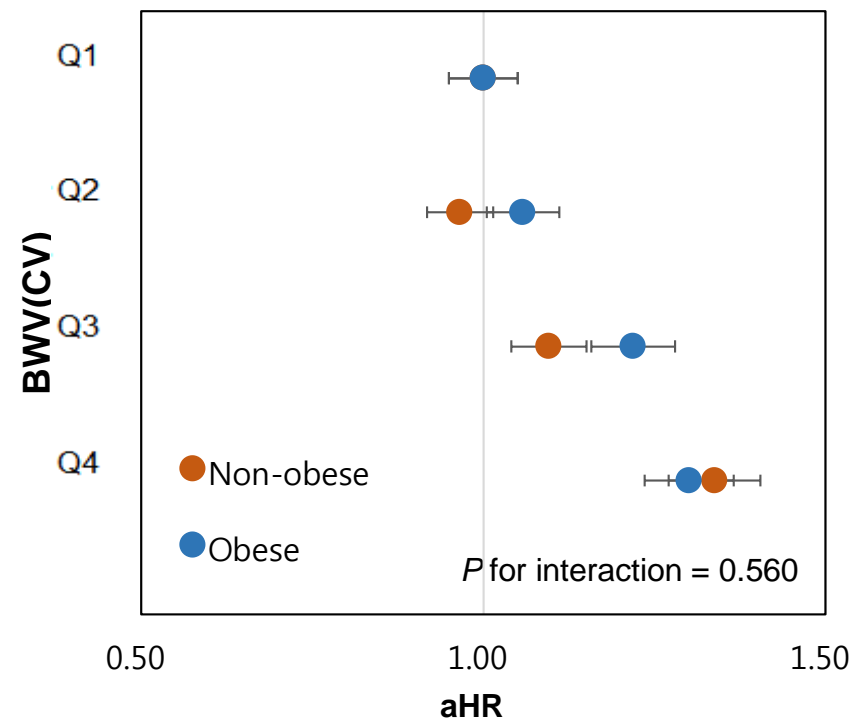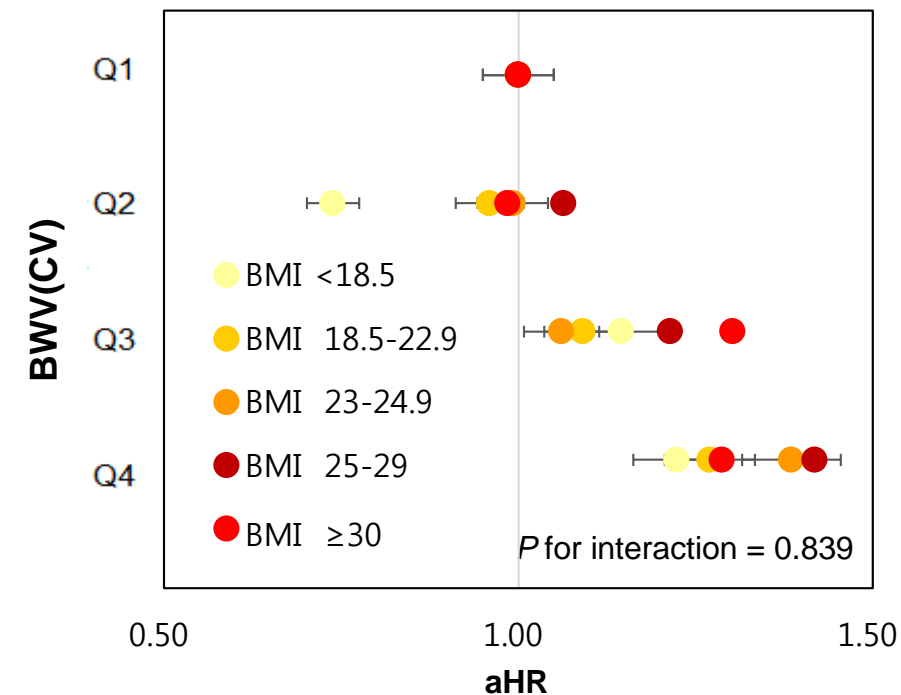

Figure S2.

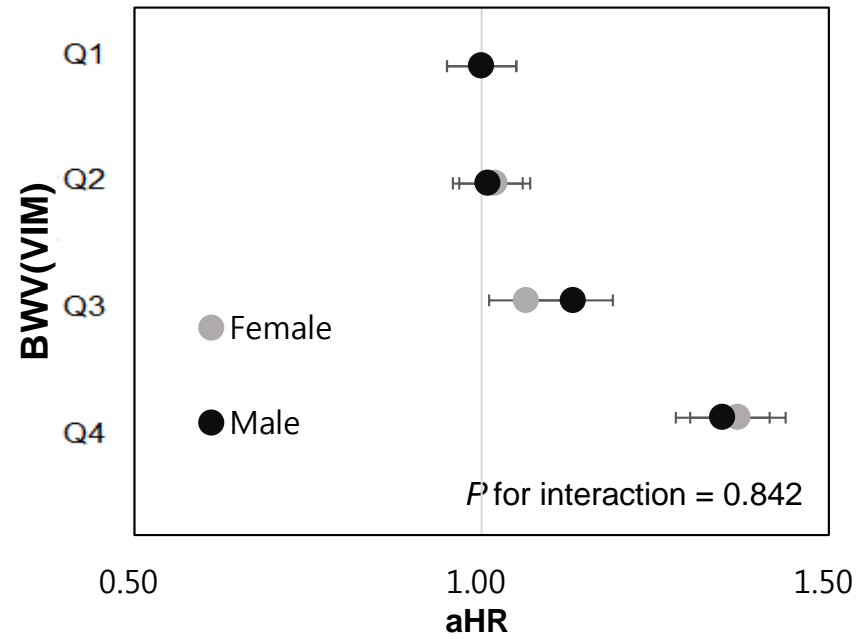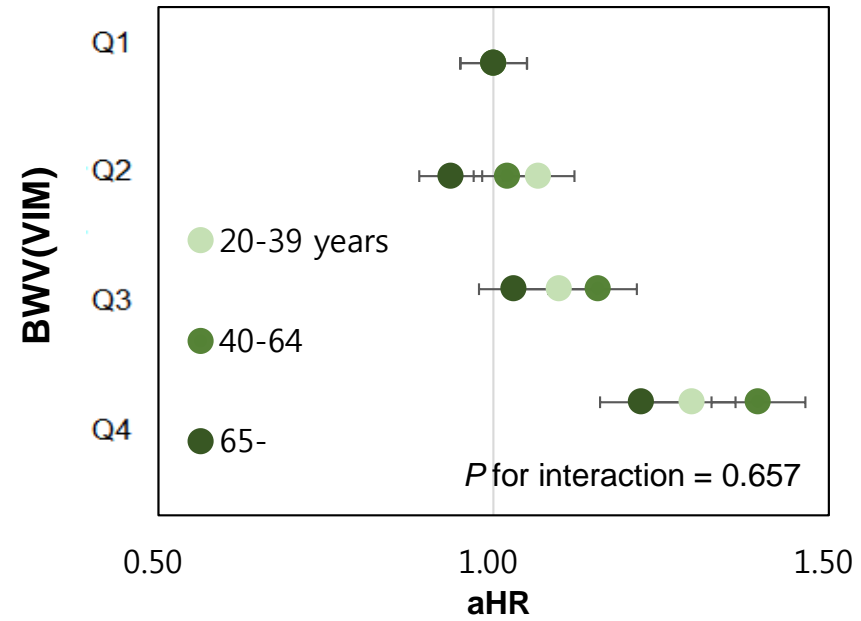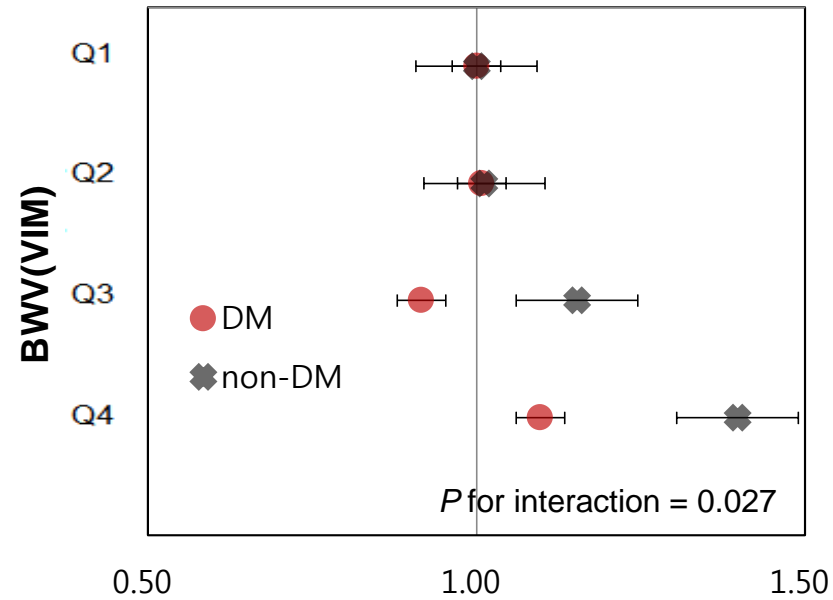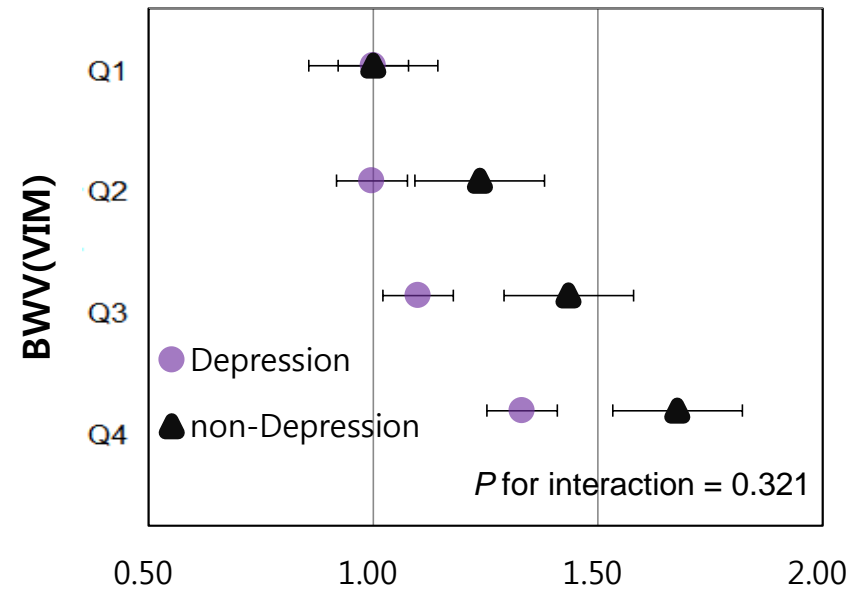

Figure S3.

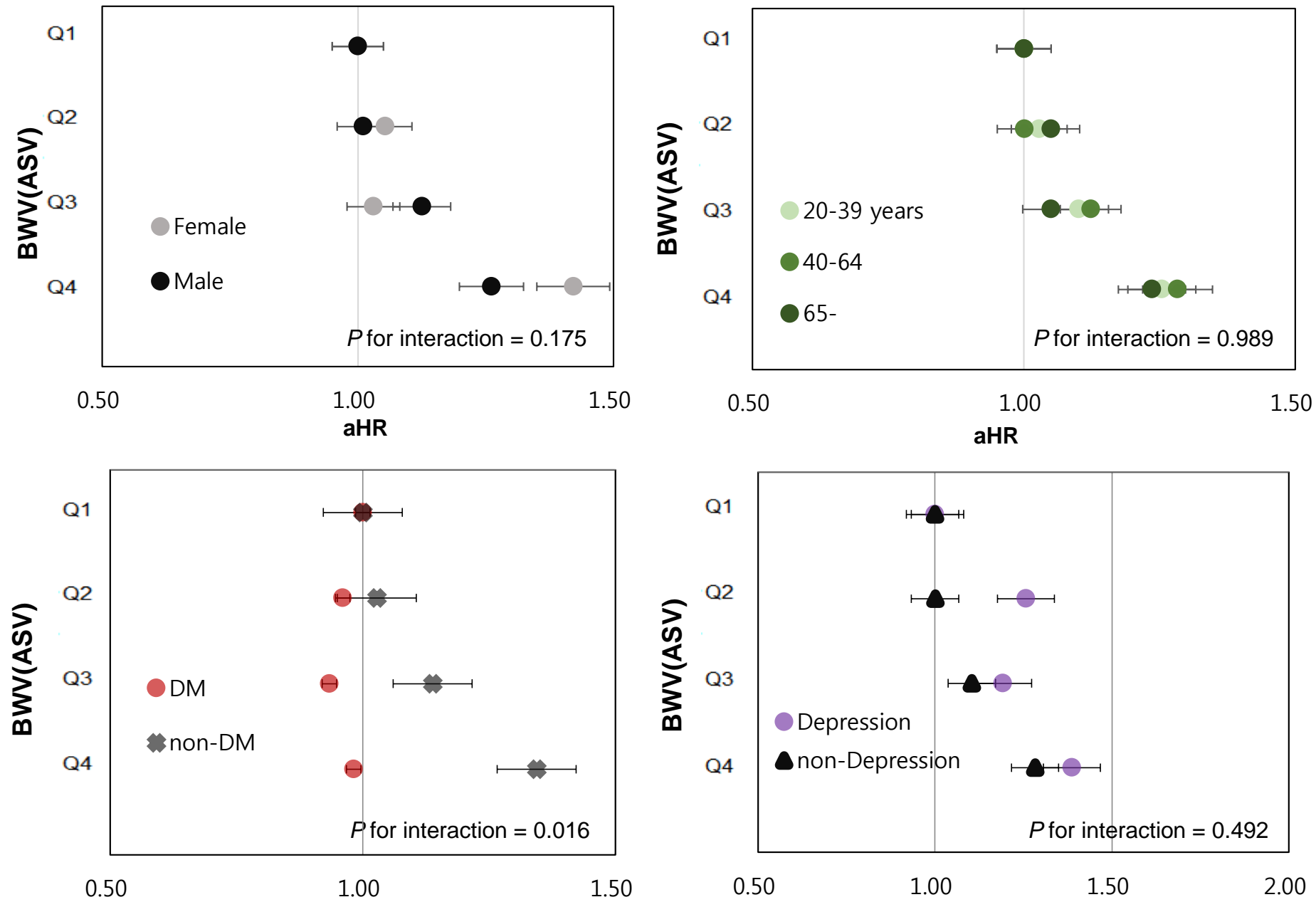

**Figure S4.**

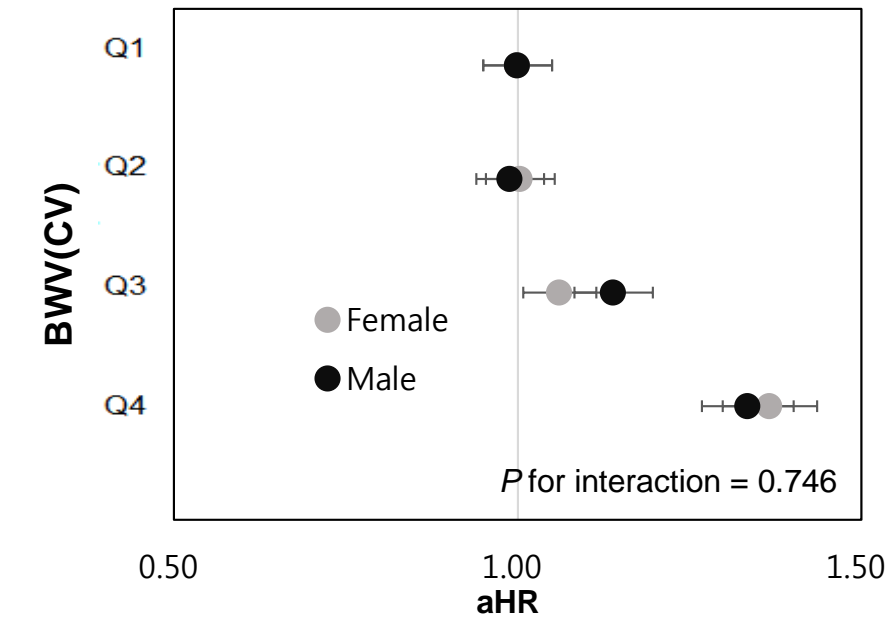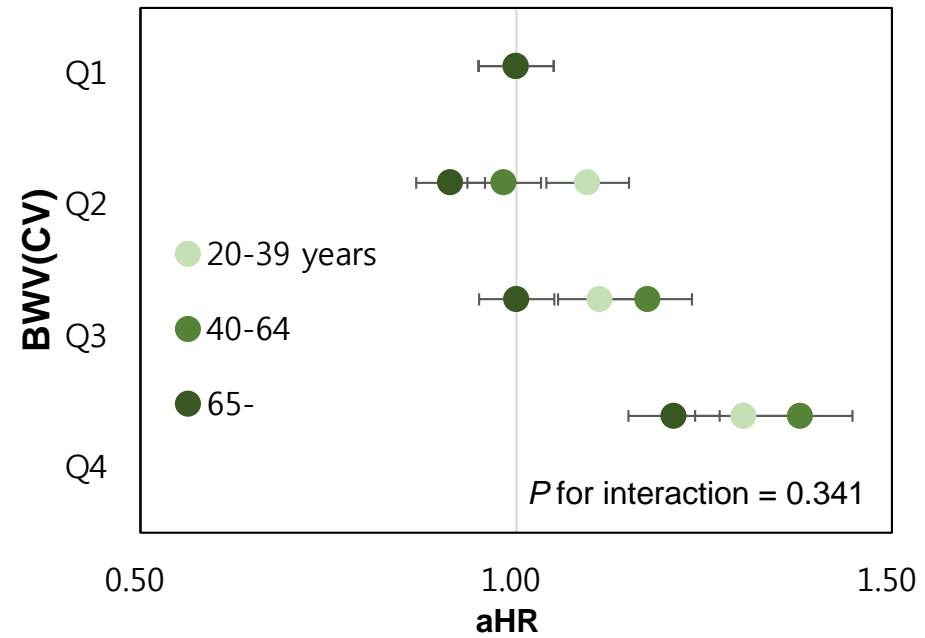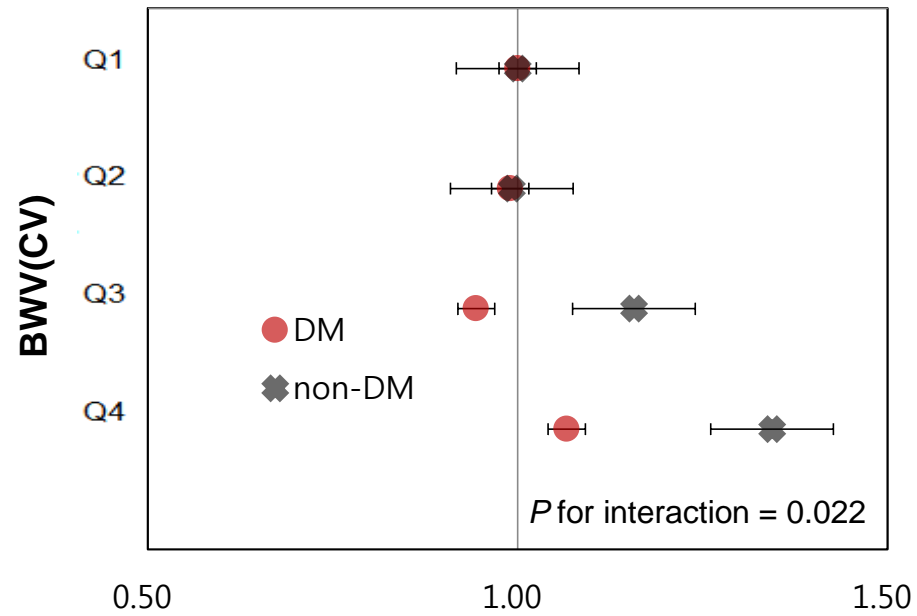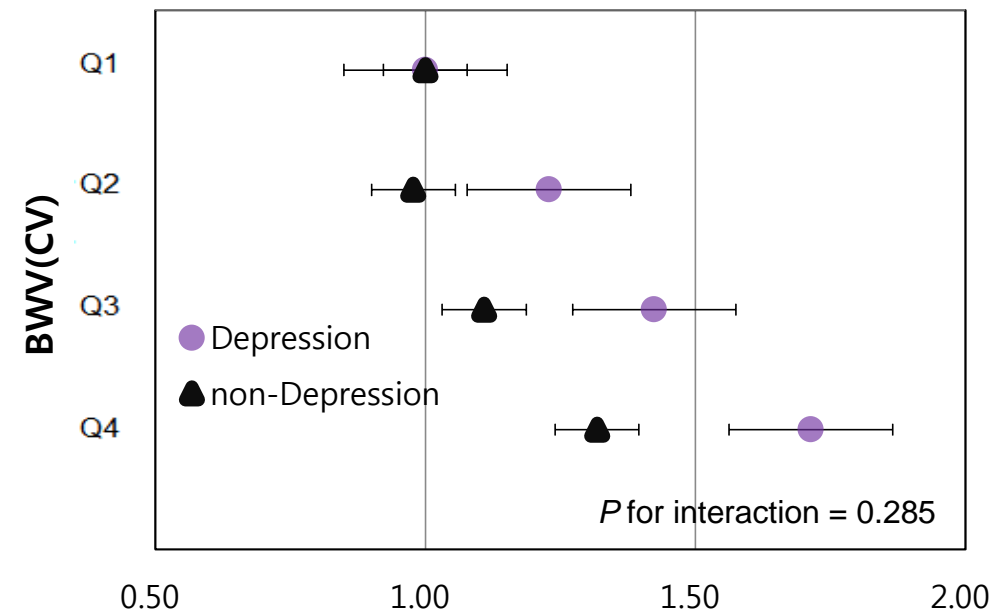

Supplement: Supplementary Materials — Figure S1 (a): the association between body weight variability (ASV) and risk of suicide death in different obesity or BMI categories. We conducted an evaluation to assess the relationship between body weight variability based on average successive variability (ASV) and the risk of suicide death in various obesity or BMI categories. Regardless of obesity, greater BWV was associated with a significantly higher adjusted hazard ratio (aHR) ([95% CI] in the Q4 BWV group, 1.27 [1.72–1.39] in nonobese vs. 1.30 [1.14–1.49] in obese participants; P for interaction = 0.257). The risk of suicide death was highest in the group of patients with a BMI of 23–25 kg/m2 (aHR [95% CI], 1.35 [1.17–1.55]). Figure S1 (b): the association between body weight variability (CV) and risk of suicide death in different obesity or BMI categories. We evaluated the connection between body weight variability, specifically focusing on coefficient of variation (CV), and the risk of suicide death across various obesity or BMI categories. Irrespective of obesity status, a higher body weight variability (BWV) demonstrated a notably elevated adjusted hazard ratio (aHR) ([95% CI] in the Q4 BWV group, 1.22 [0.86–1.74] for nonobese individuals compared to 1.40 [1.23–1.60] for obese participants; P for interaction = 0.560). Notably, the group with a BMI of 25–30 kg/m2 exhibited the highest risk of suicide death (aHR [95% CI], 1.42 [1.24–1.63]). Figure S2: the relationship between BWV (VIM) and risk of suicide death according to sex, age, DM, and depression. Figure S3: the relationship between BWV (ASV) and risk of suicide death according to sex, age, DM, and depression. Figure S4: the relationship between BWV (CV) and risk of suicide death according to sex, age, DM, and depression. The incidence rate of suicide was approximately three times higher in men than in women (IR, 0.43 vs. 0.16 per 1,000 person-years in the Q4 group). Both sexes exhibited a similar pattern of increased hazard ratio (aHR) with highe [file 7670729.f1.zip › Supplementary figures.pdf]
